# A Comprehensive Review on Deep Eutectic Solvents: Their Current Status and Potential for Extracting Active Compounds from Adaptogenic Plants

**DOI:** 10.3390/molecules29194767

**Published:** 2024-10-09

**Authors:** Malgorzata Stanisz, Beata J. Stanisz, Judyta Cielecka-Piontek

**Affiliations:** 1 Department of Pharmacology and Phytochemistry, Institute of Natural Fibres and Medicinal Plants, Kolejowa 2, PL, 62-064 Poznan, Poland; 2 Department of Pharmaceutical Chemistry, Poznan University of Medical Sciences, Rokietnicka 3, PL, 60-806 Poznan, Poland; bstanisz@ump.edu.pl; 3 Department of Pharmacognosy and Biomaterials, Poznan University of Medical Sciences, Rokietnicka 3, PL, 60-806 Poznan, Poland

**Keywords:** deep eutectic solvents, flavonoids, ginsenosides, polysaccharides, solvent extraction, active compounds, phytocompounds, adaptogens, *Panax ginseng*, *Scutellaria baicalensis*, *Schisandra chinensis*

## Abstract

Deep eutectic solvents (DESs) have attracted attention from researchers as novel compounds for extracting active substances because of their negligible toxicity, polarity, and ability to be tailored depending on the experiment. In this review, we discuss deep eutectic solvents as a promising medium for the extraction of adaptogenic compounds. In comparison to traditional methods, extraction with the use of DESs is a great alternative to the excessive usage of harmful organic solvents. It can be conducted in mild conditions, and DESs can be designed with different precursors, enhancing their versatility. Adaptogenic herbs have a long medicinal history, especially in Eastern Asia. They exhibit unique properties through the active compounds in their structures, including saponins, flavonoids, polysaccharides, and alkaloids. Therefore, they demonstrate a wide range of pharmaceutical effects, such as anti-inflammatory, antibacterial, and anticancer abilities. Since ancient times, many different adaptogenic herbs have been discovered and are well known, including *Panax ginseng*, *Scutellaria baicalensis*, and *Schisandra chinensis*. Active compounds can be extracted using standard methods, such as hydrolyzation, maceration, and conventional reflux extraction. However, due to the limitations of classical processing technologies, there has been a need to develop new and eco-friendly methods. We focus on the types of solvents, extraction efficiency, properties, and applications of the obtained active compounds. This review highlights the potential of DESs as eco-friendly alternatives for extracting bioactive compounds.

## 1. Introduction

**Figure 1:**
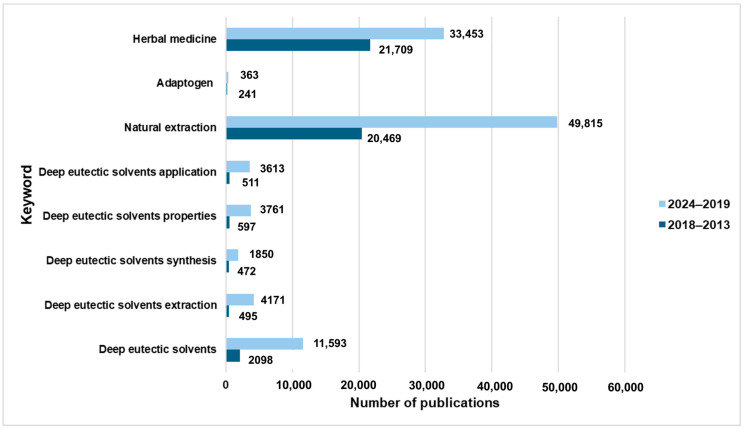
Number of publications related to selected keywords in the timeframes of 2013–2018 and 2019–2024.

Deep eutectic solvents (DESs) have been employed as reaction media for several years; however, they have garnered increased interest from researchers only recently. Consequently, there remains much to discover regarding the application of DESs as green solvents [1]. DESs offer numerous advantages, including a superior capacity to dissolve and stabilize various substances that exhibit limited solubility in traditional solvents. Furthermore, DESs can simultaneously function as catalysts and solvents, thereby enhancing the efficiency of chemical reactions and increasing both yield and selectivity [2]. Most DESs are derived from natural substances, exhibiting significant biodegradability and reduced vapor pressure, which mitigate their environmental impact. Currently, the application of DESs is predominantly restricted to laboratory-scale procedures; therefore, future research should focus on evaluating the feasibility of scaling up these processes [3]. Eutectic solvents can be tailored through the selection of precursors for their synthesis, enabling the design and customization of various DESs with specific properties, such as hydrophobicity, viscosity, polarity, and application potential. By carefully selecting the appropriate hydrogen bond acceptor (HBA) and hydrogen bond donor (HBD), researchers can enhance the selectivity of DESs for particular procedures [4,5]. As indicated by a literature review of Scopus-indexed publications between 2019 and 2024 (see Figure 1), there has been a notable increase in research focusing on the synthesis, properties, and applications of DESs when compared to the years 2014 to 2018.

Additionally, a rise in interest regarding the extraction processes utilizing DESs has been observed. Over 4000 valuable manuscripts were published between 2019 and 2024, in contrast to fewer than 500 records from 2014 to 2019. Researchers are continually exploring modifications to reactions to limit the use of toxic organic solvents, thereby improving the health of living organisms and the state of the environment. It is crucial to recycle used solvents and materials, as well as to evaluate chemical processes for enhanced efficiency. Selecting more eco-friendly solvents is a fundamental step towards achieving sustainable chemical processes. Solvents play a critical role in enhancing the selectivity and temperature of reactions, as well as dissolving, purifying, separating, and cleaning [4]. Thus, it is imperative to replace harmful solvents with safer alternatives. DESs represent a class of solvents that can be integrated as more sustainable options for the extraction of bioactive compounds. Research has indicated that traditional extraction methods often result in reduced yields, limited selectivity, as well as excessive energy and solvent consumption [6]. Notably, studies have demonstrated that adaptogens can be effectively extracted using DESs; there has been increased interest in herbal medicine, which has been used in Eastern Asia since ancient times. According to Scopus, from 2019 to 2024, more than 33,000 publications related to this topic have been published. In recent years, the world has changed drastically. People are now able to travel long distances, use smartphones, and remain constantly online. This has resulted in numerous benefits, including rapid development, increased curability of diseases, new machines that can aid survival, and the discovery of new drugs, all of which contribute to an improved overall quality of life. However, there are also some civilizational changes that negatively impact human health. Increasing industrialization, environmental pollution [7], urbanization [8], poor nutrition [9], stressful work [10] and life environments, as well as low physical activity [11] can lead to the development and occurrence of lifestyle diseases. Hypertension, heart disease, allergies, depression, autoimmune and neurodegenerative diseases, cancer, obesity, and diabetes are common lifestyle diseases that unfortunately affect a large segment of the population and are responsible for more than 80% of deaths worldwide [12]. There is an immense need to replace conventional treatments, such as taking drugs and undergoing surgery, with a more natural and gentler approach. The most important changes are lifestyle modifications, which include nutrition, stress management, social support, physical activity, and relaxation. It has been proven that the symptoms of many diseases, such as type 2 diabetes, hypertension, obesity, and coronary heart disease, can be reversed [13,14,15]. Moreover, adaptogens are valuable compounds extracted from plants that exhibit unique properties, including anti-inflammatory, anticancer, and antibacterial activities [16,17]. They can also mitigate the effects of chronic stress and help reverse the symptoms of lifestyle diseases.

Standard databases including PubMed, Science Direct, Web of Science, and Scopus were used to search the literature using the keywords, e.g., ‘adaptogens’ or ‘adaptogenic plants’ or ‘deep eutectic solvents’ or ‘*Panax ginseng* applications’. The review was prepared carefully with the guidelines for systematic reviews and meta-analysis guidelines.

Here, in this comprehensive review, we have summarized the characteristics of deep eutectic solvents, their properties, and application possibilities. We have focused on the green extraction of active phytocompounds. DESs are gaining a lot of popularity nowadays and there are many research and review articles; however, in this publication, the most recent achievements in the extraction of adaptogenic compounds with the use of DESs were presented and described. We believe that this review may be a good inspiration for other researchers to develop novel green extraction procedures using DESs and adaptogenic plants.

## 2. Deep Eutectic Solvents

Recently, DESs have become a very popular topic in research and general chemistry. The product consists of a mixture of HBDs, such as sugars, acids, alcohols, carboxylic acids, amines, and HBAs, including quaternary ammonium salts (e.g., betaine or choline chloride), which are combined together through hydrogen bonding interaction [18]. DESs can be divided into four main groups: halide and quaternary salts (type I), hydrated metal halide and quaternary salt (type II), hydrogen bond donor and quaternary salt (type III) and hydrogen bond donor and metal halide (type IV) [19]. Choline chloride is used the most frequently as an HBD, in addition to urea, glycerol, and citric acid. As a new approach, organic substances, including glucose and inorganic salts, have been used to prepare deep eutectic solvents. An HBA and HBD form one chemical compound, with non-symmetric, large ions, and low lattice energy, which results in a new characteristic of the composed substance [18].

DESs can be prepared with the use of a variety of methods including freeze-drying, grinding, and evaporation. Different types of DES preparation and its characteristics are shown in Table 1.

There are also different types of deep eutectic solvents. One of the most commonly used types is the natural deep eutectic solvents (NADESs), which are formed from naturally obtained precursors. NADESs can be obtained by mixing together HBDs, such as amino acids, sugars, and carboxylic acids with HBAs, including lactic acids, betaine hydrochloride, and choline chloride. They exhibit valuable properties, e.g., chemical and thermal stability, non-flammability, and non-volatility below atmospheric conditions with lower viscosities [23].

Another group of DESs is formed by therapeutic deep eutectic solvents (THEDESs), in which one of the components consists of an active pharmaceutical ingredient (API) and importantly is also liquid at room temperature. THEDESs can be widely used for pharmaceutical procedures such as drug development to improve the permeation, absorption, or solubility of a compound. They are prepared with the use of both synthetic polymers and also biopolymers [24].

**Figure 2:**
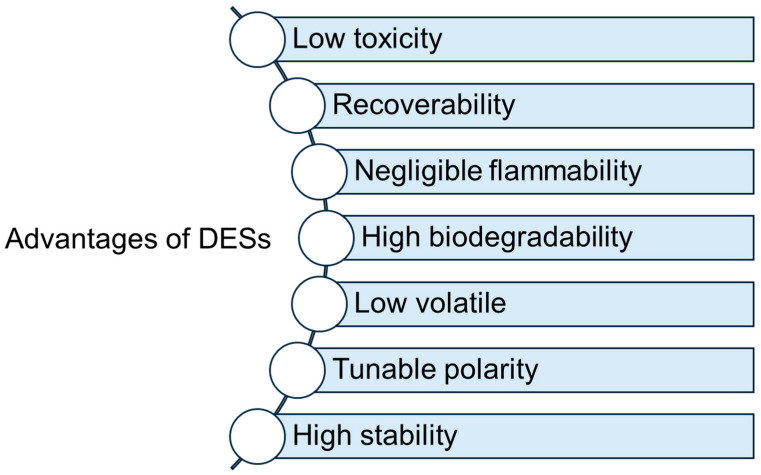
Advantages of DESs.

Polymeric deep eutectic solvents (PODESs) form another important group of DESs and they are mostly used for the delivery of active substances. They exhibit a lot of advantages, such as good biodegradability and low cost; however, they show high viscosity even showing a solid state at room temperatures [25]. Their general advantages are presented in Figure 2.

The physicochemical properties of DESs are a very valuable topic when discovering new substances. DESs can exhibit unique characteristics depending on the used precursors for their preparation. Density and viscosity are some of the most important parameters, which can dictate the application route of solution; however, surface tension, melting point, pH polarity, refractive index, and miscibility also play an important role in describing DESs [26,27]. Different properties with their descriptions are shown in Table 2.

**Figure 3:**
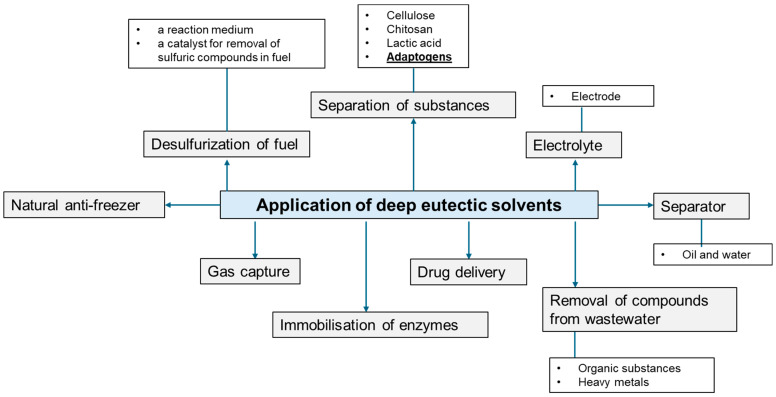
Application of DESs [20,33,34,35,36,37].

DESs can be used as media for enzymatic and chemical reactions, carriers of active compounds, antimicrobial and antioxidant agents, as well as in the extraction of active compounds from plants including oils [30,31,32]. Many different application routes are shown in Figure 3. Moreover, in Section 4 Application of Eutectic Solvents, DESs for extraction of active compounds from adaptogenic plants are closely described.

## 3. Adaptogens

Adaptogens are a highly valuable group of substances, predominantly derived from plants and, in some cases, from fungi. Consequently, they have been utilized by humans since ancient times. [38,39]. The term ‘adaptogen’ is closely related to ‘adaptation’, which is a physiological process that describes how organisms respond to environmental challenges through intercellular and extracellular interactions [40]. These interactions, recognized since ancient times, are very important in drug discovery because information about their effectiveness is based on hundreds of years of experience documented in folk medicine across different continents [41]. It has been confirmed that herbal medicine shows the ability to treat and prevent different diseases with its therapeutic effect when consumed correctly [42,43]. Active compounds promote the adaptation and survival rate of living organisms in stressful situations [44]. The stress response and remaining homeostasis are effects caused by the adaptogens, which have a wonderful regulatory effect on cellular metabolism at the molecular level [45]. The human body should be resistant to most stressors, which may be chemical, biological, psychological, or physical; therefore, adaptogens show the ability to act like stimulants to activate the metabolism and defense system, teach the body to withstand stress, as well as to reverse physical effect of constant stress to restore balance of mind and health [46]. They also should not cause any disorder in the physiological functions of organisms while restoring the proper immune response if the immune system shows poor functioning by underreacting or overreacting to certain factors. Moreover, they exhibit the power to restore the balance of brain chemistry which may affect the function, memory, and mood of an individual [47]. Adaptogens can also target several different receptors, including mineralocorticoid, progestin, estrogen, serotonin corticosteroid, and receptor tyrosine kinases as well as nicotinic acetylcholine [48]. Active phytocompounds also trigger growth factors, antioxidant enzymes, and neuropeptides; moreover, they are able to inhibit apoptosis and protect neurons through stimulation of synaptogenesis- and neurogenesis [49,50,51].

There are some requirements, which have to be fulfilled to describe an active compound as an adaptogen. Humans should not expect any harmful effects on their bodies while supplementing them. There should be only beneficial side effects, like regulated sleeping and eating schedules as well as higher energy levels. Moreover, adaptogens should also reduce unfavorable effects in stressful conditions, including fatigue, depression, and infections [52]. Importantly, they show a variety of properties, including antioxidant, anti-fatigue as well as anti-cancer protection, and act as an immunomodulant and immunostimulant. They increase physical endurance, cardiovascular protection, and radioprotective activity [16].

**Figure 4:**
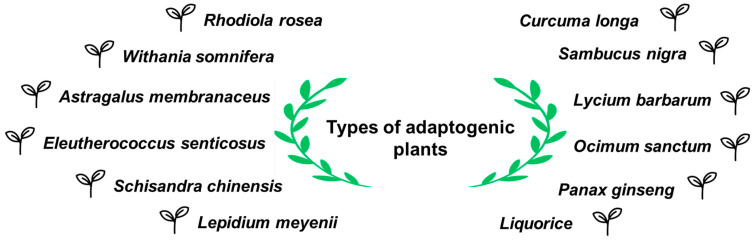
Different types of adaptogenic plants.

There are a large number of plant-based adaptogens, including *Eleutherococcus senticosus*, *Panax ginseng*, *Withania somnifera*, *Schisandra chinensis* as well as *Valeriana officinalis* (see Figure 4). The main active compounds which determine the therapeutic effect and can be isolated from plants are ginsenosides, torvanol, salidroside, rutin, a variety of polysaccharides, flavonoids, alkaloids, saponin, phytosteroids, and phytosterols [53,54].

Ginsenosides are the main active compound in Ginseng; moreover, there are also saponins, peptides, alkaloids, and lignans in its structure. Ginseng shows great anticancer, anti-inflammatory, and anti-fatigue activities. It helps to improve and promote mental health and improve the memory. Bai et al. co-fermented *Panax ginseng* with multi-enzyme-coupling probiotics and prepared an extract with probiotics and high levels of polysaccharides and ginsenosides. The obtained product was tested on mice and it was observed that their intestinal flora was stabilized, and their immune system was improved significantly [55]. In another study, An and co-workers combined Korean red ginseng with probiotics to treat diabetic wounds, which were exposed to diesel exhaust particles. It was confirmed that the prepared mixture treated wounds, especially diabetic ones, with high efficiency by controlling the enzymes and accelerating the healing time [56]. Lee et al. used Korean red ginseng as a reducing agent for the synthesis of gold bimetallic nanoparticles. The prepared particles showed great anticancer ability during chemo–PTT combination therapy and can be used as a drug delivery system for other substances [57]. Interestingly, the Trametes versicolor endophytic fungus was isolated from wild ginseng. The obtained substance was employed to produce saponins using in vitro fermentation and the total amount of substance was around 2.365 mg/mL, which was more than three times higher than with standard procedure [58]. Ginseng is a valuable plant, but it might be harder to consume because of its bitter taste. Han and co-workers loaded chitosan and gelatin particles with red ginseng extract. It was confirmed that capturing adaptogenic extract into biopolymeric particles reduced the bitter taste of the active substance and improved its acidic and thermal stability; therefore, it can be used as a food ingredient [59]. It was also shown that the rhythm of body temperature and reduction of inflammation can be improved with the supplementation of ginseng extract. Moreover, the active substance regulates the genes, which are related to inflammation, gut microbiota, and the circadian clock [60].

Another widely used plant is *Acanthopanax senticosus* (Siberian ginseng), which has the ability to prevent diseases. It consists mostly of saponins and polyphenols which enable the anti-inflammatory and antibacterial properties of the plant. The polysaccharide was extracted from *A. senticosus* and its properties of immune support and antitumor activities were examined with the use of tumor cell lines, such as Crocker sarcoma S180, hepatic carcinoma H22, and uterine cervical carcinoma U14, which were implanted in mice. It was observed that the polysaccharide showed an inhibitory effect on tumor cell lines [61]. Innovative hydrogels were prepared with the addition of *A. senticosus* and *Osmundastrum cinnamomeum*. Prepared material enhanced the homeostasis and healing of wounds and supported coagulation, which might be linked to the targeting of relevant factors by active compounds during the healing process [62]. Therefore, eleutheroside B, the most active compound in *A. senticosus*, exhibits cardioprotective effects and has the ability to suppress atrial fibrillation [63]. Fu et al. also stated that *Acanthopanax senticosus* extracts can effectively suppress the oxidative stress in the tissue of the brain; therefore, in the future, they might be used for the treatment of Parkinson’s and other diseases [64]. Extracts of *A. senticosus*, which include polysaccharides, glycosides, and flavonoids, can be also used as a food additive to enrich the rice wine to create a healthier beverage with unique flavor and bioactive components. Additionally, the extract improves the antioxidant activity of wine and enables good sensory characteristics [65].

The root of *Withania somnifera*, known also as Ashwagandha, has been widely used for many years for classical Ayurvedic formulations, and it has also been established that it exhibits anti-diabetic, anti-aging, anti-oxidant, neuro- and cardioprotective properties. Balkrishna and co-workers showed non-clinical toxicity evaluation of the whole plant of *W. somnifera*. Rats were orally exposed to the 100, 300, and 1000 mg/kg/day doses of adaptogenic extract for 28 days with 14 days of recovery and additional observation period. It was observed that *W. somnifera* is nontoxic with a dose of up to 1000 mg/kg/day for rats of either sex [66]. Jepkorir et al. used an aqueous extract from *W. somnifera* for controlling, managing, and treating rheumatoid arthritis to prevent damage to bones. The adaptogenic extract exhibited anti-inflammatory activity and, as a result, lowered the levels of pro-inflammatory cytokines with great efficiency [67]. Extracts of *W. sominfera* can also be used in the beauty sector. It was shown that the daily application of the Ashwagandha root extract in the form of a serum, for 75 days improved the health of hair and accelerated its growth; therefore it may be a safer option to use for people who are suffering from alopecia [68].

*Lycium barbarum* polysaccharide (LBP), which is widely extracted from wolfberry fruits, has been used in traditional Chinese medicine. They can be characterized by great anti-aging and anticancer as well as antioxidant and anti-inflammatory properties. Guo et al. established that medium-sized particles of LBP in ranges of 100–300 kDa showed stronger capability of induction and binding affinity than smaller particles around 10 kDa. In the gastrointestinal tract, particles tend to degrade into smaller particles; however, they still manage to promote the production of cytokine [69]. Moreover, they can also improve the function of the gut barrier and inflammatory profiles, showing their prebiotic activity as well as changes in the composition of gut microbiota [70]. It was also confirmed that *L. barbarum* polysaccharide shows great performance against lung injuries as it has the ability to suppress the inflammation caused by internal and external factors. Sewelam and co-workers found that extract from wolfberry fruits can moderate the injuries of the lung and spleen, caused by titanium dioxide nanoparticles. They prevent TLR4/NF-xB mediated injury through regulation of heme oxygenase-1 mRNA expression [71]. An interesting application approach for LBP was proposed by Liang and co-workers. They found that the polysaccharides derived from *L. barbarum* showed a lot of beneficial effects for elderly people, including increased immune-enhancing activity, proliferation ability, and increased phagocytosis capacity. The prepared extract may be suitable for addition to fluid food for elderly people who have a problem with dysphagia [72]. On the other hand, Wang et al. presented exosomes from *L. barbarum*, in which isoliquiritigenin was encapsulated and then incorporated into 3D-printed hydrogel scaffolds. The obtained material exhibited neurogenic differentiation and anti-inflammatory properties. Therefore, it was used for the regeneration of spinal cord injuries. The product promoted regeneration, the recovery of motion function, and the inhibition of inflammation; therefore, the new route of delivery of insoluble drugs via plant exosomes was promoted [73]. Interestingly, *L. barbarum* leaf flavonoids together with collagen were successfully used as a lutein emulsifier, prolonged its stability, and protected the compound from premature release and destruction, simultaneously enhancing its bioavailability [74]. Similarly, whey protein *L. barbarum* L. leaves flavonoids were also used as a functional carrier of β-carotene. The prepared emulsion showed good salt tolerance, enhanced light and thermal stability, as well as enhanced bioavailability of the encapsulated compound [75].

*Schisandra chinensis* is a very valuable plant in traditional Chinese Medicine. It contains in its structure valuable chemical compounds, including oils, polysaccharides, acids, lignans, and flavonoids. It is used in hypnosis and to protect the liver, exhibit therapeutic, antifungal, and antitumor effects, and support preventing asthma and other lung diseases. Long et al. discussed the antifungal effect of *S. chinensis*, which inhibited the growth of *Alternaria alternata* on apples, after the harvest season. The fungus is responsible for most of the losses of fruit and causes black spot disease. An ethanolic extract of *S. chinensis* fruit strongly inhibited the growth of fungus with an EC50 of 1882.00 mg/L [76]. Polysaccharides of *S. chinensis* show a great potential for the protection of nerves. Therefore, it may be useful in preventing and treating Alzheimer’s disease. They can improve the memory ability and learning skills of mice as well as moderate the damage to the neuron cells in the hippocampus and reduce the abnormally deposited Aβ-protein [77]. On the other hand, polysaccharides of *S. chinensis* have the ability to protect the kidney and liver from side effects caused by cyclosporine A, an immunosuppressive drug, prescribed after organ transplantation, which may affect important organs. The polysaccharides of *S. chinensis* improve fibrosis degree protect liver cells from function injury and promote the LX-2 cells proliferation [78]. *S. chinensis* shows not only protection on the liver but also the brain and can protect the organs from neurological and behavioral disorders which can be caused by alcohol through moderation of ethanol-induced neurotoxicity. Therefore, the water extract can also protect nerves from ethanolic damage. It was also tested on Caenorhabditis elegans worms, and it was concluded that the extract reduced the concentration of ethanol in its tissues, reduced oxidative stress, and alleviated the motility loss of organisms [79]. Interestingly, subarachnoid hemorrhage can lead to early brain injury; therefore, Jin and co-workers extracted Schizandrin A from *S. chinensis* and studied its mechanism to contain the side effects of subarachnoid hemorrhage. It was described that the presented extract alleviated inflammation and protected neurons against injury, neurological problems, and brain edema. Schizandrin A can be used as a novel and natural therapeutic compound [80]. *S. chinensis* extracts also exhibit antidepressant effects and, therefore, may affect the noradrenergic, dopaminergic, and serotonergic systems [81].

In China, there are a lot of *Sophora japonica* trees which are very beneficial in economic and ecological sectors. After blooming, flower buds become a waste, which is rich in phenols, phytoestrogens, flavonoids, and polysaccharides. Extracts show great antibacterial, antioxidant, neuroprotective, anti-inflammatory, and cardiovascular properties. They are able to protect the organisms against the development of neurological disorders and support the enhancement of quality of life. A methanol extract of the flower buds of *S. japonica* can prevent infiltration of immune cells and epithelial hyperplasia. Moreover, it also shows the potential to treat contact dermatitis [82]. *S. japonica* hydroalcoholic extracts support resistance to oxidative stress, neuroprotective activities, and antioxidant properties; therefore, they may be used to treat the diseases of the central nervous system as well as Huntington’s, Alzheimer’s, and Parkinson’s diseases [83]. Interestingly, it was discussed by Zheng et al. that *S. japonica* extracts can moderate symptoms such as bloody stools, inflammation, tissue damage, and colon shortening through the regulation of lipid metabolic pathways and, therefore, also support the inhibition of the progression of ulcerative colitis to colitis-associated colon cancer [84]. On the other hand, dried *S. japonica* flower buds were used to extract rutin. The obtained active compound was then combined with β-cyclodextrin to form a complex with enhanced fluorescence intensity. The complex was applied as a selective and sensitive sensor to detect copper ions with high efficiency [53].

Another widely used East Asia plant is *Scutellaria baicalensis*, which contains active compounds including baicalin, baicalein, and wogonin. It exhibits anti-inflammatory and antibacterial properties against, e.g., *Streptococcus mutans* and *Porphyromonas gingivalis*. Zheng et al. prepared novel nanofibrous wound dressings, which were loaded with baicalin as an antibacterial component for the acceleration of wound healing. Moreover, the active compound improved the hydrophobicity of the prepared membrane and, therefore, it was able to permeate from the nanofibers into the wound [85]. In another study, Paczkowska-Walendowska et al. prepared 3D-printed chitosan-based hydrogels, which were loaded with the *S. baicalensis* extract. The active substance was in amorphous form; therefore, an increase in the release of baicalin was confirmed and the active substance could support and accelerate the healing of periodontal wounds [86]. Baicalin can also be effectively used as an anti-diabetic agent. It was observed by Nagarajan et al. that dosing 50 mg/kg of baicalin orally to albino male rats reduced the levels of glucose and glycated hemoglobin in the blood, thereby enhancing the levels of insulin secretion and hemoglobin. Moreover, it also protected the renal tissue with great anti-diabetes management and prevention of diabetic nephropathy [87]. Huang et al. prepared functional granules with Eudragit S100 and baicalin with pH-sensitive properties to target the colon. It was observed that targeted delivery of active compound improved the therapeutic effect in colitis with a reduction of expression levels of tumor necrosis factor alpha with the increase in superoxide dismutase activities. It was concluded that baicalin also showed potential application for ulcerative colitis [88]. Interestingly, the baicalin can also be used in treatment against SARS-CoV-2 while targeting the human angiotensin-converting enzyme II protein. It was reported by Lin and co-workers that the baicalin inhibited the infection by 98% [89].

As was presented in this part, there are many different adaptogenic plants, in which active substances can be used in various industrial sectors (see Figure 3). There is also a concern with its extraction, which is crucial in terms of the stability and bioavailability of phytocompounds. There are many different technologies for the extraction of active phytocompounds from plants or mushrooms. The first group consists of standard extraction methods, including hydrolyzation, maceration, infusion, Soxhlet, or conventional reflux extraction. Most of these procedures are simple and low-cost; however, most of them require the usage of a lot of solvents, high temperatures, and time, which might not be beneficial for the procedure. There are also more novel extraction methods, which include supercritical fluid extraction, ultrasound-, enzyme- and microwave-assisted extractions, subcritical-water extractions, as well as high hydrostatic pressure assisted procedures. These methods are mostly eco-friendly, with lower temperatures and shorter timeframes, but some of them might be more expensive to perform and lead to changes in the structure of the extracted active compound. One of the newest extraction methods is the use of DESs as a reaction medium for the extraction procedure.

## 4. Application of Deep Eutectic Solvents

### Extraction of Adaptogens with Deep Eutectic Solvents

DESs have been recently used to extract active substances from adaptogens. It is a fairly novel, green, and efficient approach, which may in the future enable to endow or completely replace standard organic solvents.

Tu et al. used deep eutectic solvents for the extraction of polar ginsenosides from *Panax ginseng* with the assistance of ultrasound extraction. For this procedure, eighteen different DESs were created, but during the experiments, it was concluded that the most effective substance was the one obtained with choline chloride and urea in the proportion of 1:2. The authors determined that the optimum reaction conditions for extracting ginsenosides were 15 mL/g of liquid and solid ratio content, 15 min of an ultrasonic extraction time, and 20 wt% of DES water content. The extraction process was highly efficient and around 31% higher than for standard 70% ethanol [90]. Similar conclusions were made by Zhou and co-workers. In their research, *P. ginseng* was also used as a model compound to extract ginsenosides and polysaccharides without the use of organic substances and it was confirmed that the extraction rate was significantly higher than standard solvent extraction. Moreover, they combined different DESs with aqueous two-phase systems. The samples were ultrasonicated for 30 min and the extraction was performed at 60 °C with water content of 20%. The best results were obtained for DESs prepared with ethylene glycol and choline chloride [54]. In another research, Liu and co-workers used a natural deep eutectic solvent to extract active compounds from the leaves and stems of *P. ginseng*. It was also confirmed that the best results were obtained with 20% of water content. The highest yields of ginkgolides, phenolics, and ginsenosides were obtained with the use of malic acid and glucose (1:1) DES. Surprisingly, during the DES extraction lower amounts of toxic ginkgolic acids were detected than for standard solvents, therefore the extract may be in the future used for pharmaceutical applications [91]. Jeong et al. also extracted polar ginseng saponins from white ginseng with the use of DES composed of glycerol, L-proline, and sucrose (9:4:1) with great efficiency and bioactivity against HTC-116 cancer cells. Interestingly, they also performed the recycling of deep eutectic solvents through freeze-drying of solution. It was confirmed that the same DES, after the recovery process, can be used up to 3 times with great extraction efficiency, up to 83% [92].

Polysaccharides can be extracted from *A. senticosus* using deep eutectic solvents. Xue et al. used a DES with a ratio of 4:1 L-proline to L-malic acid for efficient extraction of active compounds with extraction rate of 35.452 ± 0.388 mg/g, which is a higher efficiency than for hot water extraction. The extraction conditions included a solid-to-liquid ratio of 31.068 g/mL with a water content of 32.364%, at 60 °C of extraction temperature. The polysaccharides exhibited high antioxidant activity, low molecular weight, and great anti-glycation activity [93]. Interestingly, polysaccharides extracted with the use of DESs can also be used as an immunomodulator or feed additive in broilers to proliferate the beneficial bacteria and reduce the number of harmful ones. Moreover, polysaccharides increased the immune organ index and restored the growth performance of IL-2 and IFN-γ and immunoglobulin IgG1 levels in broilers [94]. On the other hand, Zhang and co-workers extracted flavonoids from *A. senticosus* using glycerol and levulinic acid (1:1) deep eutectic solvent with a water content of 28%, extraction temperature and time of 55 °C and 73 min, respectively. It was observed that the total flavonoid yield reached 23.928 ± 0.071 mg/g, which was 40.7% higher than for standard ultrasonic-assisted ethanol extraction. Moreover, DESs can be also reused twice for effective and environmentally friendly extraction of flavonoids [95]. Yu et al. extracted lignin from *A. senticosus* residue with the use of four different methods, including milled wood, alkali, ethanol, and DES methods. However, the highest content of lignin was obtained using the DES method, the biopolymer showed the lowest UV-resistant activity; therefore, it may be less suitable for suncream application, but may find its usage in electrochemistry or medicine [96]. Shi et al. extracted saponins, phenylpropionc acid, and terpenoids from *A. senticosus* root with the use of choline chloride and lactic acid-based DESs, and more than 90 different phytochemicals were identified with more than 15 new compounds identified in the plant’s root. The extraction of active compounds with the use of deep eutectic solvents is a promising method to discover new compounds, which could be found in plants and afterward used in health and food industries [97]. Mu et al. presented the extraction procedure of eleutheroside E from *A. senticosus* using two different deep eutectic solvents. It was discussed that the extraction rate was up to 6 times higher than for the standard ethanol extraction method. Eleutheroside E has a significant therapeutic effect in the treatment of central and cardiovascular diseases [98].

*Schisandra chinensis* is another valuable adaptogenic plant, in which active compounds can be extracted with the use of deep eutectic solvents. Li and co-workers used choline chloride and ethylene glycol (1:3) DES for the simultaneous extraction of polysaccharides and essential oils from *S. chinensis* using a synergic microwave and ultrasound extraction method. Polysaccharides and essential oils were extracted with the efficiency of 8.56 g/100 g and 12.2 mL/kg, respectively. It was concluded that the active compounds obtained from adaptogenic plants can be used as natural antioxidants [99]. An innovative approach was developed by Liu and co-workers, in which DESs were used as a delivery vehicle. As a model lipophilic compound *S. chinensis* extract was used for its deposition into the hydrogel beads. The advantage of this method is that there is an elimination of organic solvents because the deep eutectic solvent has the ability to diffuse from the polymer carrier after the delivery of active molecules. The use of DES may in the future improve the bioavailability of valuable lipophilic molecules, which have the ability to reduce inflammation and cancer possibility [100]. On the other hand, Yan et al. prepared green temperature-responsive DESs and applied them to extract lignanoids and polysaccharides from *S. chinensis*. The separated active phytochemicals showed strong antioxidant activity; moreover, DESs can be recycled up to four times and still perform extraction of multi-polar components with high efficiency [101]. In another study, Chen and co-workers extracted five lignans including Schizandrol A (10.89 mg/g), Schizandrol B (8.616 mg/g), Schisantherin A (4.019 mg/g), Schisandrin A (4.893 mg/g), and Schizandrin B (5.318 mg/g) from *S. chinensis*. The deep eutectic solvent consisted of glycolic acid and choline chloride with a molar ratio of 1:4 and 30% of water content. The extraction time and temperature were 20 min and 70 °C, respectively. The results showed that the presented extraction method with the use of the DES is eco-friendly and cheap [102].

Meng et al. separated polysaccharides from *A. membranaceus* var. *Mongholicus* using deep eutectic solvent, which consisted of choline chloride and oxalic acid (1:2 molar ratio). They stated that the optimal extraction parameters were as follows: 54 min of ultrasonic irradiation, 50 °C of ultrasonic temperature, and liquid to solid ratio of 24 mL/g. The extraction efficiency of polysaccharides was 61.4 ± 0.6 mg/g with high purity, without any impurities including proteins, pigments, and nucleic acids [103].

Yu and co-workers presented the extraction of carotenoids and their esters from *L. barbarum* fruit using the deep eutectic solvent extraction method. The best results were obtained using a DES which consisted of choline chloride and malonic acid. The seventeen different carotenoids with inhibitory activity and high yields were identified. Extracted active substances can prevent and treat benign prostatic hyperplasia. Moreover, the method is environmentally friendly in comparison to standard solvent extraction [104]. In another research, Tang et al. prepared a temperature-switchable deep eutectic solvent to extract the polysaccharides from *L. barbarum*. As an optimal extractant, the DES consists of tetracaine and lauric acid (1:1, molar ratio). The reaction condition was as follows: 70 min of reaction time, 35 °C of extraction temperature, and 70 wt% concentration of DES with maximum extraction efficiency of 45 mg/g. The prepared temperature-switchable DES can be regenerated and reused up to five times with a high extraction yield of 80.2%. Extracted active compounds showed good scavenging activity against model free radicals [105]. On the other hand, Ali et al. used a deep eutectic solvent composed of choline chloride and *p*-toluene sulfonic acid with the assistance of ultrasound extraction to obtain flavonoids from *L. barbarum* fruits. It was shown that this green procedure was more efficient than the standard extraction procedure with high extraction yields for morin (12.7 mg/g), rutin (9.1 mg/g), and myricetin (57.2 mg/g) with overall flavonoid recovery of 75.6–96.9% [106]. Recently, Gu and co-workers have presented the extraction of rhamnogalacturonan-I pectin with the use of a mixture of choline chloride and propylene glycol (1:2 molar ratio) deep eutectic solvent with 20% water content. The extraction yield of polysaccharides was higher for DES extraction than for the traditional procedure; moreover, the neutral sugar and uronic acid contents were obtained through this green procedure. It was also observed that the extracted pectin was characterized by great prebiotic activity [107].

Rutin can be also extracted from *Sophora japonica* buds using a deep eutectic solvent consisting of chlorine chloride and triethlene glycol (1:4 molar ratio). The best extraction efficiency of 279.8 mg/g was achieved under the conditions of 28.3 min and 70 °C of extraction time and temperature, respectively. Moreover, water content was around 20% with a solid and liquid ratio of 10 mg/g. Extracted active compounds exhibited great antioxidant activity and were tested against free radicals [108]. Zhao et al. also used the same deep eutectic solvent to extract rutin from *S. japonica*. The DES contained 20% of water and the extraction efficiency of 194.17 ± 2.31 mg/g was obtained [109]. Le et al. extracted rutin from *Sophora japonica* using a mixture of choline chloride and ethylene glycol with extraction and recovery yields of 26.20% and 94.9%, respectively. The deep eutectic solvent can be recycled and recovered up to three times without any loss of extraction efficiency. It was also shown that rutin extracted with the use of the DES exhibited higher antioxidant activity than the one obtained with standard solvents and showed no cytotoxic effect against the human embryonic kidney cell line [110]. On the other hand, Zhang and co-workers synthesized rutin using a natural deep eutectic solvent to hydrolyze it into quercetin through enzyme degradation. Rutin was up to 3116 times more soluble in DES than in water and, therefore, the extraction procedure was more efficient, biodegradable, and eco-friendly. The best extraction efficiency of active compounds was obtained using a DES composed of chlorine chloride and glycerol at a molar ratio of 1:1 [111]. In another study, Ni et al. prepared quercetin extracts from *S. japonica* using acidic natural deep eutectic solvents, consisting of choline chloride and citric acid. It was stated that the acidity of the hydrogen bonding donor is crucial for the deglycosylation of rutin and that the preparation of rutin is dependent on the temperature of the procedure. Both quercetin extracts and DESs showed low cytotoxicity to Caco-2-cells, which directly depended on the amount of citric acid [112].

Sohail and Ahmed compared the two most commonly used extraction techniques, i.e., ultrasound and heat-assisted extractions to obtain bioactive substances from *W. somnifera* roots. Sodium acetate and glycol deep eutectic solvents were used. They tested the prepared extracts for total phenolic content, radical scavenging, and iron chelating activity as well as total flavonoid content. It was concluded that the procedure of extracting active substances with ultrasound-assisted extraction is shorter in time and more efficient [113].

**Figure 5:**
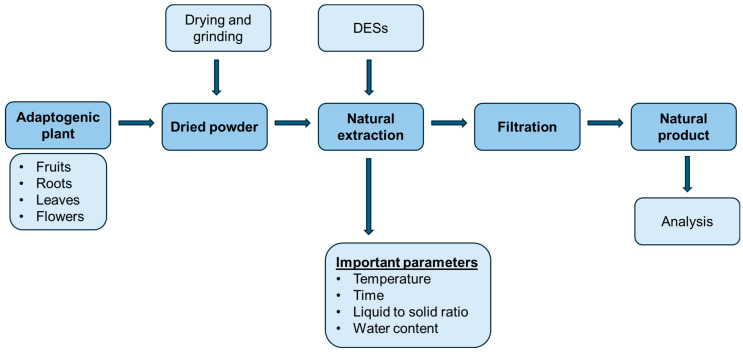
Extraction steps of adaptogens using DESs.

Baicalin can be extracted from *S. baicalensis* using deep eutectic solvent extraction method. Hydrophobic DES was used with the assistance of microwave extraction. The maximum yield of the active compound of 106.96 mg/g with a water content of 33 vol% and was similar to the standard procedure described in pharmacopoeia. The novel method may be more eco-friendly and an excellent alternative for the extraction of active compounds [114]. In another study, baicalin was extracted with the use of DES and ultrahigh-pressure extraction. The choline chloride and lactic acid (1:1 molar ratio) DES was used with 40 vol% of water content. High pressure of 400 MPa with an extraction time of 4 min was employed to obtain baicalin with a maximum yield of 116.8 mg/g, which is more effective than the traditional extraction procedure [115]. On the other hand, Wang et al. used ternary deep eutectic solvent to synthesize molecularly imprinted polymers which were decorated with Fe_3_O_4_ particles to separate the baicalein from *S. baicalensis*. It was confirmed that the proposed medium showed great selectivity and binding capacity towards baicalein. Therefore, the recovery of active phytocompound ranged from 91.6% to 99.3% [116]. Oomen et al. extracted flavonoids including flavonoid aglycones—baicalein, wogonin, oroxylin A, and scutellarein—as well as their glycosides baicalin, wogonoside, oroxyloside, and scutellarin from *S. baicalensis* stem bark. As an extraction medium, natural DESs consisting of citric acid combined with glucose, xylitol, proline, and *β*-alanine were used, with a water content of (20% to 60% *w*/*w*). It was shown that, despite the high hydrophilicity of natural DES, more hydrophilic glycosides were extracted with lower efficiency than their aglycones. Baicalein, wogonin, oroxylin A, and scutellarein were extracted up to six times higher than for the methanol standard extraction. On the other hand, baicalin, wogonoside, and oroxyoside were extracted up to 1.8 times higher than for traditional procedures. DESs can be used to improve the extraction yield of a variety of compounds with different hydrophilicity [117]. A simple, rapid, reproducible, and efficient extraction of flavonoids from *S. baicalensis* radix was presented by Hao and co-workers. As a medium for extraction, betaine and acetic acid (1:4 molar ration) deep eutectic solvent with a water content of 40% exhibited the highest yields for baicalin, baicalein, wogonoside, wogonin, and oroxylin A extracts. As it was compared to the traditional procedure with 70% ethanol as a solvent, it was stated that the extraction efficiency was higher with shorter times of procedure. The obtained active compounds exhibited great anti-inflammatory properties [118]. Xiong et al. have also presented the green and efficient extraction of flavonoids (scutellarin, baicalin, wogonoside, baicalein, and wogonin) from *S. baicalensis* using glycerol and L-proline (4:1, molar ratio) and acquired great extraction efficiency [119]. A simple, fast, and eco-friendly extraction of flavonoids from *S. baicalensis* was proposed by Wang et al. They used a switchable deep eutectic solvent composed of N,N-dimethylethanolamine and heptanoic acid (1:1, molar ratio) with homogenous liquid-liquid microextraction. Six flavonoids with different polarity including scutellarin, baicalin, scutellarein, wogonoside, baicalein, and wogonin were extracted with good efficiency [120]. Guo and co-workers also stated that the use of natural DESs for the extraction of flavonoids from *S. baicalensis* enables a shorter extraction time with higher efficiency and lower organic solvent usage compared to traditional methods. In the experiments, a deep eutectic solvent consisting of L-proline and urea (2:1, molar ratio) with water content of 40% was used to extract flavonoids with supers antioxidant activity [121]. The most important parameters of extraction with the use of DESs are presented in Table 3. Moreover, the essential steps of extraction of adaptogens are shown in Figure 5.

## 5. Conclusions

This review has provided various insights into adaptogens, their natural extraction, and the properties of active substances. Adaptogens have been well known for many centuries, particularly in East Asia. Plants that contain adaptogenic substances possess a unique ability to adapt and survive in stressful conditions. The developed range of properties, including anti-inflammatory, antibacterial, anticancer, and antitumor abilities, enables the application of active substances in various industrial fields, including medicine. They are primarily utilized as therapeutics, supporting the organism and prolonging life. Additionally, they exhibit antidepressant and anti-anxiety properties, as well as the ability to stimulate the brain and prevent memory loss. Furthermore, adaptogenic compounds have a significant impact on cardiovascular diseases, diabetes, and neurodegenerative disorders. At least 70 different types of plants can be considered adaptogenic, including *P. ginseng*, *W. somnifera*, *S. chinensis*, and *L. barbarum*. Adaptogenic compounds can be extracted from plants using various traditional methods, including conventional reflux extraction, infusion, and maceration. Although these methods are relatively inexpensive, they require substantial energy and the use of organic solvents, which may be harmful to the environment. In this context, more environmentally friendly extraction methods have begun to be developed. One such method is the use of DESs as a medium for extraction. DESs offer numerous advantages; they can be easily tailored for specific applications, and their toxicity is negligible. In this review, the extraction of adaptogenic compounds using DESs has been described, and it was concluded that the extraction efficiency is at least the same—if not higher—compared to standard methods. We hope that this review will mark the beginning of further exploration of this subject by other researchers.

Recently, interest in the use of natural medicines has increased, driven by the rising prevalence of lifestyle diseases, declining health, increased stress, lack of sleep, and poor eating habits. Doctors and researchers have begun to seek new remedies in response to the growing incidence of cardiovascular diseases, obesity, diabetes, and insulin resistance. For many years, adaptogenic active substances have contributed to preventing and reversing the harmful effects associated with recent lifestyle choices. There is a growing interest in purchasing supplements that may help improve insulin sensitivity in tissues.

Despite the numerous advantages of using herbal medicine, several topics require further discussion in future research. There is a common belief that natural products cannot be harmful; however, herbal medicines can have effects on the body and may produce side effects if not ingested properly. Before using natural substances, individuals should consult their healthcare providers, particularly those who are taking other medications, are pregnant or breastfeeding, or are children and the elderly. The most significant risks arise from taking natural supplements alongside important medications, as this may reduce the efficacy of treatment and lead to unexpected side effects. It is also essential to purchase herbal products from reputable and legitimate sources to minimize the risk of acquiring unlicensed or contaminated products, which may contain harmful substances not listed on the label. Greater emphasis should be placed on regulating and testing the composition of herbal supplements. Nonetheless, using herbal substances with caution can be highly beneficial for human health. There remains much to explore in researching the mechanisms and applications of adaptogenic plants, but we believe that our comprehensive review will serve as a solid foundation for future essential research. While the incorporation of DESs into adaptogenic extraction can be very beneficial because of their eco-friendliness, higher extraction efficiencies, mild extraction conditions, and versatility, there are also some limitations and challenges which should be resolved. DESs exhibit promising results on a laboratory scale, but the transition to industrial-scale extraction processes can be problematic. The viscosity of DESs can complicate large-scale applications resulting in reduced efficiency during processing. Moreover, some of the solvents can decompose at higher temperatures, which could lead to limitations in their use in processes that require elevated temperatures. New regulatory evaluations should also be issued in terms of the usage of the new solvent systems. While comparing DES extraction with traditional methods, it can be observed that DESs show typically higher extraction efficiency, that can be achieved in shorter timeframes, because of their ability to solubilize a wide range of compounds. On the other hand, traditional solvents can more often be available and cost-effective; however, while some components during the preparation of DESs can also be inexpensive, the optimization and tailoring of solvents may result in increased overall costs. DESs are also generally considered environmentally friendly, but additional assessments are necessary to confirm their sustainability. Moreover, DESs can also be reused multiple times; however, their effectiveness may decrease with each cycle. Therefore, careful optimization for sustainability is required. DESs offer many promising advantages over traditional solvent extraction methods; however, addressing their limitations and challenges is crucial for their successful implementation on a larger scale. Continued research and development can also improve specific areas, including process optimization, comprehensive characterization of solvents, cost analysis, and industry regulations.

This review is expected to stimulate the rapid advancement of green chemistry and generate new ideas for the further development of extraction techniques and applications of adaptogenic active substances. Adaptogenic plants and their therapeutic potential offer a variety of possibilities for further research. However, some topics need additional investigation. First of all, sustainable, eco-friendly extraction methods which minimize solvent use and environmental impact should be prioritized. Novel extraction methods should not only enhance the yield and purity of active substances but also support global sustainability goals. Research should also focus on the potential synergistic effects of combining multiple adaptogens and examine whether specific combinations can enhance efficacy and reduce side effects. It is also important to study how variations in climate, soil health, and cultivation methods affect the efficacy of adaptogenic plants. Specific requirements can lead to better agricultural practices to maximize the therapeutic potential. Another important step while developing the therapeutic effect of adaptogens is to conduct well-designed clinical trials with a focus on establishing effective dosages, long-term safety, and therapeutic chronic fatigue. There may be many plants with therapeutic effects which still need to be explored. The conduction of comprehensive studies across ecosystems would be a great start to uncover and document lesser-known adaptogens. It can also be beneficial to collaborate with the community to share their traditional knowledge about local plants. By pursuing these topics, the scientific community can contribute to a deeper understanding of adaptogenic plants and their extraction with DESs, fostering innovation, improving health outcomes, and promoting sustainable practices.

## Figures and Tables

**Table 1:** Different synthesis methods of DESs [20,21,22].

Synthesis Method	Characteristic
Heating and stirring	simple method; compounds are heated and mixed together under constant stirring for around 8 to 12 h with a magnetic stirrer; homogenous liquid is formed; synthesis is performed at temperature ranges from 50 to 100 °C; higher temperatures might lead to degradation of final products due to esterification reaction;
Freeze-drying	components are mixed first with water, then freeze-dried; and finally, the water is sublimated;
Evaporation	dissolution of compounds forming a DES and then evaporation of water at 50 °C; the substance should be placed in a desiccator;
Grinding	the compounds are mixed at room temperature, crushed with a pestle in a mortar; homogenous liquid is formed;
Ultrasound-assisted synthesis	very fast preparation of deep eutectic solvents; homogenization process for around 1 min and then the mixture is sonicated for 30 min; the steps are repeated several times to obtain a homogenous final product, which has to be stored in a desiccator at room temperature; HBD and HBA components interact through the cavitation effect, which was caused by ultrasonic waves;
Microwave-assisted synthesis	homogenization in vortex for approx. 1 min; mixture is treated in a microwave reactor at 850 W for 45 min with 600 rpm steering speed and at 80 °C; short synthesis time through dielectric heating and interaction with materials and dipole rotation; molecules of HBD and HBA components start to collide and result in fast and efficient synthesis;

**Table 2:** Characteristic of DESs [28,29].

Property	Characteristic
Melting point	the melting point of a DES is lower than the melting point of components through strong interaction between the HBD and HBA; significant impact of anion on the melting point; depends on the change of entropy, the interaction of hydrogen bond donor with hydrogen bond acceptor and lattice energies;
pH	pH is correlated with the temperature of a DES; with an increase in temperature, the pH of the deep eutectic solvent decreases linearly; the most important physical property of a DES; pH is influenced by the acidity and basicity of hydrogen bond acceptor and hydrogen bond donor;
Surface tension	surface tension is dependent on the intermolecular interactions between precursors; it varies with molar ratio, temperature, and the type of HBA and HBD; with the increase in the alkyl chain length of the cation, the surface tension of DES decreases; with the increase in organic salt, the hydrogen bond network can be disrupted and the surface tension also decreases;
Polarity	most of the DESs are polar; polarity increases with the increased interaction between hydrogen bonding donor and hydrogen bonding acceptor;
Density	only hydrophobic DESs show the densities lower than water; the reduction of density can occur with increasing temperature because of increasing ionic motion; it is dependent on the HBA and HBD molar ratio and the existence of holes and vacancies within the deep eutectic solvent; the density can be decreased due to the increase in the alkyl chain of cation resulting in the increase in free volume;
Viscosity	one of the most important factors—temperature. The breakdown of the hydrogen bond network between the HBD and HBA; with higher temperature, and lower viscosity; factors, which influence the viscosity: temperature, molar and mass ratios, and the nature of hydrogen bond acceptor and hydrogen bond donor; with higher viscosity the mobility in the small volume is lower; important are also the interactions including van der Waals and electrostatic forces between the HBD and HBA;
Refractive index (RI)	refractive index allows the information about the composition of deep eutectic solvent; the RI is higher due to larger sizes of molecules; decrease of RI results in less dense samples; there is a reduction of hydrogen bond interaction with the increase in temperature and the RI is inversely proportional to this parameter;
Conductivity	due to high viscosity, DESs exhibit low conductivity at room temperature; conductivity is dependent on the temperature, the alkyl chain length of the cation, and the molar ratio of the HBD and HBA; conductivity is strongly connected to the temperature; with higher temperatures, the ionic mobility increases, and the hydrogen bond network gets raptured;
Toxicity	DESs are mostly considered green solvents; the toxicity of DESs depends on its chemical structure and the precursors of the mixture; the acidity of DESs leads to more cytotoxic substances; moreover, the toxicity is correlated with the organic acid hydrogen bond donor, which has higher toxicity than different HBDs; the toxicity and cytotoxicity of bacteria, fungi, and viruses have been shown in several research;
Effect of water	there is a low possibility of drying DESs, because of their hygroscopic nature; physicochemical properties and biocompatibility are related to the water content and water addition to DESs and the polarity as well as solubilization capacity are influenced by the addition of water; the increased chain length of carboxylic acid results in the increased adsorption capacity and rate of water molecules adsorbed from air; dissolution of DESs in water increases the toxicity of the mixture; with the increase in water content, the parameters of the melting point, density, and viscosity are decreased by disruption of hydrogen bonds; moreover, there is an increase in ionic mobility;
Biodegradability	bacteria and fungi easily metabolize the DESs due to neutrality of hydrogen bond donors and acceptors; the most biodegradable donors are amines and the least ones are acids;

**Table 3:** Extraction parameters with the use of DESs and adaptogenic plants.

Plant	Active Substance	DESs	Water Content (wt%)	Liquid/Solid Ratio (mL/g)	Extraction Amount (mg/g)	Ref.
*P. ginseng*	Ginsenosides	Choline chloride–urea (1:2)	20	15	11.41	[90]
White ginseng		Glycerol–L-proline–sucrose (9:4:1)	-	-	8.24	[92]
*A. senticosus*	Polysaccharides	L-proline:L-malic acid (4:1)	32	31	35.45	[94]
	Flavonoids	Glycerol–levulinic acid (1:1)	28	18	23.93	[95]
*S. chinesis*	Polysaccharides	Chlorine chloride–ethylene glycol (1:3)	43	30	85.60	[99]
	Schizandrol A	Glycolic acid–chlorine chloride (1:4)	30	20	10.89	[102]
	Schizandrol B				8.62	
	Schisantherin A				4.02	
	Schisandrin A				4.89	
	Schizandrin B				5.32	
*A. membranaceus*	Polysaccharides	Choline chloride–oxalic acid (1:2)	55	24	61.4	[104]
*L. barbarum*	All-trans-β-carotene	Choline chloride–malonic acid (1:1)	-	-	9.98	[104]
	All-trans-β-zeaxanthin				5.98	
	Β-cryptoxanthin monopalmitate				55.34	
	Zeaxanthin monopalmitate				44.99	
	Polysaccharides	Tetracaine–lauric acid (1:1)	70	25	465.00	[105]
	Morin	Choline chloride–*p*-toulene sulfonic acid	-	-	12.70	[106]
	Rutin				9.10	
	Myricetin				57.20	
*S. japonica*	Rutin	Choline chloride–thiethylene glycol (1:4)	18	10	279.80	[108]
			2	-	194.17	[109]
		Choline chloride–glycerol (1:1)	20	-	291.57	[111]
		Choline chloride–citric acid (1:1)	10	-	10.10	[112]
	Quercetin				38.70	
*W. somnifera*	Flavonoids	Sodium acetate–glycerol (1:3)	50	-	6.08	[113]
*S. baicalensis*	Baicalin	Decanoic acid–tetrabutylammonium chloride (1:2)	33	16	106.96	[114]
		Choline chloride–lactic acid (1:1)	40	110	116.80	[115]
		Citric acid–*β* alanine (1:1)	40	-	39.40	[117]
	Baicalein				2.70	
	Scutellarein				7.50	
	Wogonin				18.60	
	Wogonoside				59.40	
	Oroxylin A				2.90	
	Oroxyloside				5.40	
	Baicalin	Citric acid–proline (1:1)	60	-	32.00	
	Baicalein				3.20	
	Scutellarein				4.60	
	Wogonin				10.90	
	Wogonoside				82.40	
	Oroxylin A				7.00	
	Oroxyloside				12.00	

## Data Availability

Not applicable.
